# Regulation of pathological blood-brain barrier for intracranial enhanced drug delivery and anti-glioblastoma therapeutics

**DOI:** 10.32604/or.2022.025696

**Published:** 2022-10-10

**Authors:** KAI WANG, FENGTIAN ZHANG, CHANGLONG WEN, ZHIHUA HUANG, ZHIHAO HU, YUWEN ZHANG, FUQIANG HU, LIJUAN WEN

**Affiliations:** 1 Key Laboratory of Prevention and Treatment of Cardiovascular and Cerebrovascular Diseases of Ministry of Education, Gannan Medical University, Ganzhou, 341000, China; 2 College of Pharmaceutical Sciences, Zhejiang University, Hangzhou, 310058, China; 3 Department of Orthopedics, First Affiliated Hospital of Gannan Medical University, Ganzhou, 341000, China; 4 Medical College of Soochow University, Suzhou, 215123, China; 5 Department of Infectious Diseases, Ganzhou People’s Hospital, Ganzhou, 341000, China; 6 Ganzhou Key Laboratory of Neuroinflammation Research, Gannan Medical University, Ganzhou, 341000, China

**Keywords:** Blood-brain barrier, Physiological, Pathological, Glioblastoma, Intervention

## Abstract

The blood-brain barrier (BBB) is an essential component in regulating and maintaining the homeostatic microenvironment of the central nervous system (CNS). During the occurrence and development of glioblastoma (GBM), BBB is pathologically destroyed with a marked increase in permeability. Due to the obstruction of the BBB, current strategies for GBM therapeutics still obtain a meager success rate and may lead to systemic toxicity. Moreover, chemotherapy could promote pathological BBB functional restoration, which results in significantly reduced intracerebral transport of therapeutic agents during multiple administrations of GBM and the eventual failure of GBM chemotherapy. The effective delivery of intracerebral drugs still faces severe challenges. However, strategies that regulate the pathological BBB to enhance the transport of therapeutic agents across the barrier may provide new opportunities for the effective and safe treatment of GBM. This article reviews the structure and function of BBB in physiological states, the mechanisms underlying BBB pathological fenestration during the development of GBM, and the therapeutic strategies of GBM based on BBB intervention and medicinal drugs transporting across the BBB.

## Introduction

The leading cause for the failure of tumor chemotherapy is the lack of effective drug delivery in the lesion site, which is still a severe challenge for glioblastoma (GBM) therapy [1,2]. The increase in tumor resistance to drugs primarily lies in the use of low-specific and highly cytotoxic therapeutics and fluctuations in plasma drug levels. The blood-brain barrier (BBB) is a specialized endothelial structure that is partially covered by pericytes and almost entirely surrounded by the end feet of astrocytes. The primary function of BBB is to maintain the normal physiological part of the brain and the balance of the central nervous systems (CNS) environment [3,4]. It is noteworthy that the lack of fenestration in endothelial cells results in the low permeability of BBB, and tight junctions (TJs) between adjacent brain endothelial cells make the barrier 50–100 times tighter than peripheral microvessels. Except for some special transport channels, almost 100% biological macromolecules and 98% small molecules cannot cross the BBB into the brain, which seriously limits the efficacy of therapeutic agents for brain diseases, including GBM [5]. Therefore, the critical scientific problem to be urgently solved for treating GBM is to improve the transport efficiency of therapeutics into the brain effectively.

Recently, extensive and substantial progress has been made in GBM therapy, including brain receptor-mediated targeted therapy [6], focused ultrasound opening the BBB [7] and Borneol for “orifice-opening” of the BBB [8]. Among them, the preferred strategy for GBM-targeted treatment is receptor-mediated intracerebral transport which shares characteristics of high specificity, affinity and selectivity [9,10]. Several studies have described that many kinds of receptors are overexpressed on the BBB, including insulin receptors (IR) [11], low-density lipoprotein receptors (LDLR) [12], transferrin receptors (TfR) [13] and nicotinic acetylcholine receptors (nAChRs) [14]. And their ligands are usually employed to facilitate receptor-mediated intracerebral delivery of anti-GBM therapeutic drugs. However, the intracerebral transport efficiency of medications is still unsatisfactory. In the author’s crude opinion, some of these reasons are mainly attributed to the fact that current studies on brain-targeted drug delivery systems primarily focus on the interactions between ligands and their receptors, as well as targeted cells and targeted tissues, while ignoring the dynamic changes of BBB function during the development and treatment periods of brain diseases. One or even several treatments of the brain-targeted delivery system may achieve tumor-inhibiting effects in the short term. Still, in the long run, its impact on the restoration of pathological BBB can continue to inhibit the intracerebral delivery efficiency of therapeutic drugs, and lead to the failure of their treatment. In addition, the poor druggability of the vast majority of brain-targeted drug delivery systems limited their clinical application. Therefore, there is still a long way to find safe and effective ways to go to treat brain diseases, and anti-GBM therapy still needs the discovery of new mechanisms, the breakthrough of new technologies and the emergence of new treatments.

The pathological process of GBM is likely to be closely related to BBB, which might also play a vital role in the occurrence and treatment of GBM [15]. Under physiological conditions, BBB is a biological membrane barrier composed of brain microvascular endothelial cells and their TJs, basement membranes and the end feet of glial cells around capillaries [16]. TJs structure is an essential morphological basis of the BBB, forming the low permeability of the barrier and its high resistance properties [17]. Under pathological conditions, the occurrences of brain diseases [18–21], including GBM, Parkinson’s disease (PD), cerebral ischemia and Alzheimer’s disease (AD), are usually accompanied by pathological impairment of BBB, with decreased TJs proteins and increased BBB permeability.

Studies have confirmed that BBB pathological fenestration provides an opening paracellular pathway for the transport of therapeutics across the BBB. Hu et al. have demonstrated that BBB pathological fenestration in GBM provided an extra paracellular route for angiopep-2 modified glycolipid-like nanoparticles transporting across the barrier *in vitro* and *in vivo* [22]. Nevertheless, during multiple dosing treatments for GBM, the first administration with brain-targeted nanotherapeutics would cause pathological BBB restoration, which resulted in poor intracerebral transport efficiency during re-administration and eventual treatment failure. Our previous study has confirmed that the treatment of targeted Ap-CSssSA/P nanoparticles could restore pathological BBB mainly via VEGF downstream signaling pathway inhibition. However, pathological BBB functional recovery seriously reduces the intracerebral transport efficiency of Ap-CSssSA/P nanoparticles, leading to a poor outcome in GBM therapy. Therefore, combined sequential treatment with functionalized Ap-CSssSA/P nanoparticles and BBB permeability regulator SC79, which could reversibly re-open the restored BBB, was proposed to treat GBM. And the strategy finally produced an observable and significantly enhanced anti-GBM effect *in vivo*, compared to single Ap-CSssSA/P nanoparticles treatment [23]. Hence, the regulation of pathological BBB for improving the transport of therapeutic agents across the BBB may provide new opportunities for GBM effective treatment.

## Physiological Characteristics of the BBB

**Figure 1:**
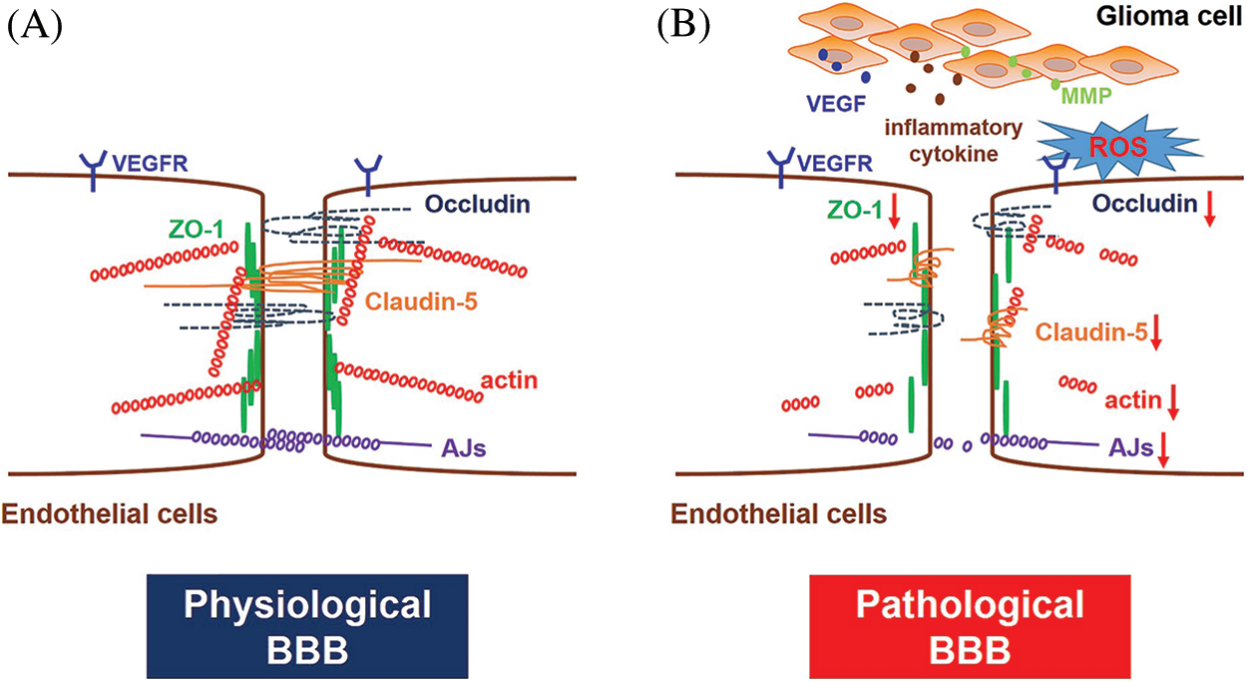
BBB structure under physiological and pathological conditions. (A) Under physiological conditions, the BBB is formed by specialized endothelial cells characterized by no fenestration and extensive TJs and AJs. Transmembrane proteins of TJs mainly include Claudins, Occludin and ZOs. TJs and AJs are generally linked to the actin cytoskeleton. (B) GBM growth promotes the over-secretion of VEGF, inflammatory cytokines, ROS and MMPs in the lesion area, which may cause down-regulated expression of TJs and AJs proteins and cytoskeleton disruption, ultimately leading to BBB disruption and pathological fenestration.

BBB is the most distinct structural difference between the peripheral and cerebral vasculatures, and the barrier achieves CNS homeostasis by strictly regulating molecules transport between brain parenchyma and blood [24]. The BBB is formed by brain endothelial cells and is characterized by extensive windowless TJs, cytoskeleton and adherens junctions (AJs) (Fig. 1A).

TJs, distributed at the apex of cerebral endothelial cells, are consist of a series of paralleled and interconnected chains of transmembrane and cytoplasmic proteins that resemble an intricate network [25]. It is often thought that the closed endothelial fissures of the TJs allow the continued formation of vascular structures. The molecular biology of the TJs is quite complicated, and there are mainly three proteins in TJs, including Claudins, Occludin and Zonula Occludens (ZOs) proteins. Claudins homotypically bind to other Claudin molecules in adjacent cerebral endothelial cells, generating the primary seal of TJs [26,27]. Among Claudins, Claudin-5 is usually considered a critical marker of TJs breakdown and BBB pathological fenestration. The growth of GBM reduces expression and alters the intracellular distribution of Claudin-5, which in turn lead to BBB functional disruption [28]. Occludin is not necessary for TJs formation, and its function is mainly an additional support structure for TJs regulation as indicated in the knockdown and knockout experiments [29,30]. The regular expression and localization of other junctional proteins, such as vascular endothelial cadherin, are well compensated for the loss of Occludin [31]. Submembranous TJ-related proteins, also known as peripheral occlusive domain proteins, like ZO proteins, are another component of TJs. They might be involved in forming scaffolds that connect TJs and cytoskeleton [32,33]. Moreover, other junctional adhesion molecules present in brain endothelial cells, including JAM-A, JAM-B and JAM-C, are also involved in the formation and maintenance of TJs [17,34], as well as in the establishment of cell polarity and the promotion of leukocytes migrating across endothelial junctions [16]. In addition, the brain endothelial cytoskeleton, composed of three critical elements: actin microfilaments, intermediate filaments and microtubules, is also vitally crucial in the integrity establishment of TJs [35].

Adherens junctions (AJs) are distributed beneath the TJs in the basal region of the lateral plasma membrane. AJs are mainly composed of transmembrane glycoproteins, whose primary function is to connect cytoplasmic proteins to the cytoskeleton and exert additional contractile structures between adjacent endothelial cells to maintain the stability of the BBB [32]. Moreover, AJs also participate in brain endothelial cell interactions, cell polarity initiation and paracellular permeability regulation [36].

## Mechanisms of BBB Pathological Disruption in GBM

BBB breakdown is generally considered detrimental in most cases because it leads to an influx of partial blood components, including leukocytes, potentially neuroactive compounds, and water (edema). The leakage of harmful substances into the brain would also accelerate the deterioration of the disease, leading to a poor prognosis of the disease. Abnormal increase of vascular endothelial growth factor (VEGF), reactive oxygen species (ROS), inflammatory cytokines and matrix metalloproteinases (MMPs) during the growth period of GBM, could seriously cause BBB disruption, accompanied by TJs proteins and AJs proteins down-regulation and cytoskeleton damage (Fig. 1B).

### Vascular endothelial growth factor

Recent data indicated that VEGF, an angiogenic growth factor known to be overexpressed in GBM, promoted the endocytosis of endothelial cell adhesion molecule VE-cadherin and the down-regulation of TJs proteins (including Claudin-5, Occludin, ZO-1), resulting in the disruption of BBB function and increased endothelial permeability [37,38]. Moreover, the exogenous VEGF treatment may also induce TJs structural fracture and Occludin loss, accompanied by the phosphorylation of Occludin Ser490, resulting in vascular impairment [39]. Yang et al. indicated that VEGF could increase the permeability of BBB in GBM by a paracellular pathway (down-regulation of Claudin-5 and Occludin) and a transcellular pathway (up-regulation of caveolin-1 and caveolin-2), and cGMP/PKG/NF-κB signaling pathway might also be involved in VEGF-mediated BBB disruption [40]. Inhibition of the VEGF/VEGFR signaling pathway promotes the restoration of pathological BBB, accompanied by increased expression of TJs, including Claudin-5 and Occludin, and cytoskeletal remodeling [23,41].

### Oxidative stress

Proinflammatory status, causes oxidative stress and increases endothelial expression of cell adhesion molecules. ROS are generally overexpressed in tumor cells, including GBM cells, compared to healthy cells. Studies have shown that the VEGF secreted by GBM can activate its downstream signaling pathways, which cause the increase of nitric oxide (NO) release and ROS production and finally lead to the pathological disruption of BBB and the development of brain edema [42,43]. Elizabeth et al. proposed that the generation of ROS could enhance the tyrosine phosphorylation of VE-cadherin and β-catenin, which ultimately influences AJs integrity [44]. Gerty et al. further demonstrated that ROS regulates BBB integrity, characterized by rearrangement of cytoskeleton and disappearance of TJs proteins. And this process involves specific signaling pathways, including RhoA, PI3K kinase and PKB signaling [45].

### Inflammation

In the regulation of BBB permeability, inflammatory mediators are known modulators. In most neuroinflammatory disease states, including GBM, compromised TJs, are prominent hallmark [46]. Magnetic resonance imaging (MRI) has fully verified that BBB disruption is associated with the development of vasogenic edema in patients with intracranial tumors [47]. Consistently, studies based on the GBM-bearing mice model indicate the down-regulation of TJs proteins (including Claudin-5, Occludin and ZO-1) in microvasculature may be induced by cytokines, such as interleukin-6 (IL-6), tumor necrosis factor-alpha (TNF-α), interferon-γ and NF-κB [48,49].

### Matrix metalloproteinases

As previously mentioned, increased levels of MMPs have also been observed in GBM, with disrupted basal lamina proteins and degraded TJ complexes. Zinc-containing enzymes MMPs mediates the degradation of protein substrates mainly via Zn^2+^-mediated activation of the site-bound water molecules. The accumulation of zinc in microvessels of GBM could activate MMP-2 and MMP-9, producing the remodeling and degradation of TJs [50,51]. Suppression of MMP-2/9 using SB-3CT could partially alleviate the disruption of TJs [52].

In conclusion, the pathological process of GBM causes pathological fenestration of BBB, which is rooted in the destruction of TJs structure. Therefore, signaling pathways in which TJs proteins (Occludin, Claudins and ZOs) are involved may be potential targets for BBB intervention in treating GBM.

## Targeting Pathological BBB for GBM Therapy

### Active targeting

In general, water-soluble molecules are transported across the BBB by simple diffusion through the paracellular pathway [53]. Small-molecule lipid-soluble substances such as steroids and ethanol are transported into the brain through the transcellular diffusion pathway. In addition, the transport of substances across the BBB also includes absorptive-mediated transport (AMT), carrier-mediated transport (CMT), receptor-mediated transport (RMT), active efflux transport, and cell-mediated transport [54,55]. Among them, AMT has poor selectivity, and cationic protein can bind to any negatively charged cell membrane component. In addition, the use of cationic proteins may produce potential toxicity and immunogenicity. The transport of CMT is substrate selective, and only drug molecules structurally similar to endogenous carrier substrates can be captured and transported into the brain. Previous studies have confirmed that in anti-GBM therapy, RMT is considered one of the most mature strategies for intracerebral drug delivery [56–58]. Brain endothelial cells express many receptors, including TfR, IR, LDLR, scavenger receptor, acetylcholine receptor, leptin receptor, etc. Structural modification with corresponding ligands (such as transferrin, monoclonal antibody OX26, CDX, RVG29, TGN, angiopep-2, etc.) [59–61] can promote the transport of therapeutics across the BBB via the RMT pathway, offering the possibility for GBM-targeted therapy.

### Cell penetrating peptide

Cell-penetrating peptides (CPPs) are a class of short peptides with amino acids less than 30. They are extracted from insect, viral and mammalian proteins that can induce translocation of bioactive macromolecules to cross cell membranes without interacting with receptors [62]. They are generally classified into three categories based on their physical and chemical properties: cationic, amphiphilic, and hydrophobic. Cationic CPPs are mainly composed of positively charged amino acids, such as arginine and lysine, which can interact with the negatively charged plasma membrane [63]. The apical surface of brain capillaries is densely covered with negatively charged polysaccharide-protein complexes, making positively charged CPPs effective drug carriers for brain diseases passing through the BBB. However, the low tissue specificity, poor instability, and potential cytotoxicity of CPPs *in vivo* limit their application.

### Membrane-mediated targeting

In recent years, a new class of targeted biomimetic drug delivery systems coated with biological membranes has attracted wide attention from researchers due to its immune escape and homotypic binding capacities. Various membranes have been taken for coating, such as erythrocyte [64], platelets [65], stem cells [66], tumor cells [67], bacteria [68], etc. By translocation of membrane onto the surface, these membrane-coated drug delivery systems integrate the biogenicity of membrane proteins and the chemical stability of synthetic drug delivery systems, with the advantages of good biocompatibility, low immunogenicity, and homologous targeting function at lesion sites [69,70]. Gboxin-loaded mesoporous silicon nanoparticles coated with a mixed membrane composed of erythrocyte and tumor cell membrane can effectively target glioma cells and inhibit tumor cell proliferation [71].

In addition, exosome-based technology has also been extensively studied as a means of delivering therapeutic drugs into the brain. As a kind of secretory vesicle, exosomes inherit some receptors on the surface of the cell membrane, possess the targeting ability of parental cells, and can cross a variety of biological barriers [72]. Using tumor-associated macrophage exosomes as a carrier to wrap thermosensitive liposomes could effectively target glioma cells, and photothermal combined with chemotherapy could reverse drug resistance of glioma [73]. Functionalized modification of exosomes with angiopep-2 to wrap the anti-tumor drug docetaxel can significantly enhance intracerebral drug delivery and improve its anti-glioma efficacy [74].

As a collection of natural and synthetic entities, membrane carriers show substantial therapeutic advantages and biocompatibility, a boon for researchers. However, each membrane has its advantages and limitations. For example, the erythrocyte membrane can prevent phagocytosis by the reticuloendothelial system (RES) and prolong the circulation time *in vivo*, but it lacks active targeting ability; leukocytes have homologous targeting capabilities, but are specific for certain tumors; platelets can target tumor and injured areas, but can also activate the immune system; cancer cells can attach homologously and aggressively, but have a short circulation time in the blood; stem cells have the ability to target tumors, but with low specificity; bacterial membranes have immune-inducing properties, but the process of membrane extraction is complicated due to the need to remove peptidoglycan [75–77]. Therefore, a fusion of two membranes can be effectively carried out to form a hybrid membrane with therapeutic benefits for both cells. Although significant achievements have been made in the research of membrane-coated nanoparticles, there are still some challenges to be solved that the level of translation from laboratories to clinical trials has not yet been fully developed, and more practical explorations are needed in the future.

### Blood brain tumor barrier (BBTB) targeting

The structure of BBTB is similar to that of BBB, and the barrier is located between GBM tissue and microvessels formed by brain microvascular endothelial cells. BBTB is composed of highly specialized endothelial cells, and restricts the paracellular transport of most hydrophilic molecules to tumor tissues. With the development of GBM, tumor cells invade the surrounding normal brain tissue, and when tumor volume is >2 mm^3^, BBB function and integrity are destroyed, and BBTB begins to appear. The formation of BBTB is critical for the supply of oxygen and nutrients to tumor cells’ growth [78]. Moreover, with the emergence of BBTB, GBM cells can migrate along the newly formed blood vessels to surrounding healthy brain tissue. It has been shown that efflux proteins such as P-gp, MRP1, MRP3, and BCRP overexpressed in TJs and BBTB severely limit the effective delivery of therapeutic drugs to GBM lesions [79]. Hence, the use of receptors highly expressed on BBTB, such as epidermal growth factor receptor (EGFR) and integrin receptor, may become one of the effective strategies for GBM targeted therapy.

## Opening Pathological BBB for GBM Therapy

**Figure 2:**
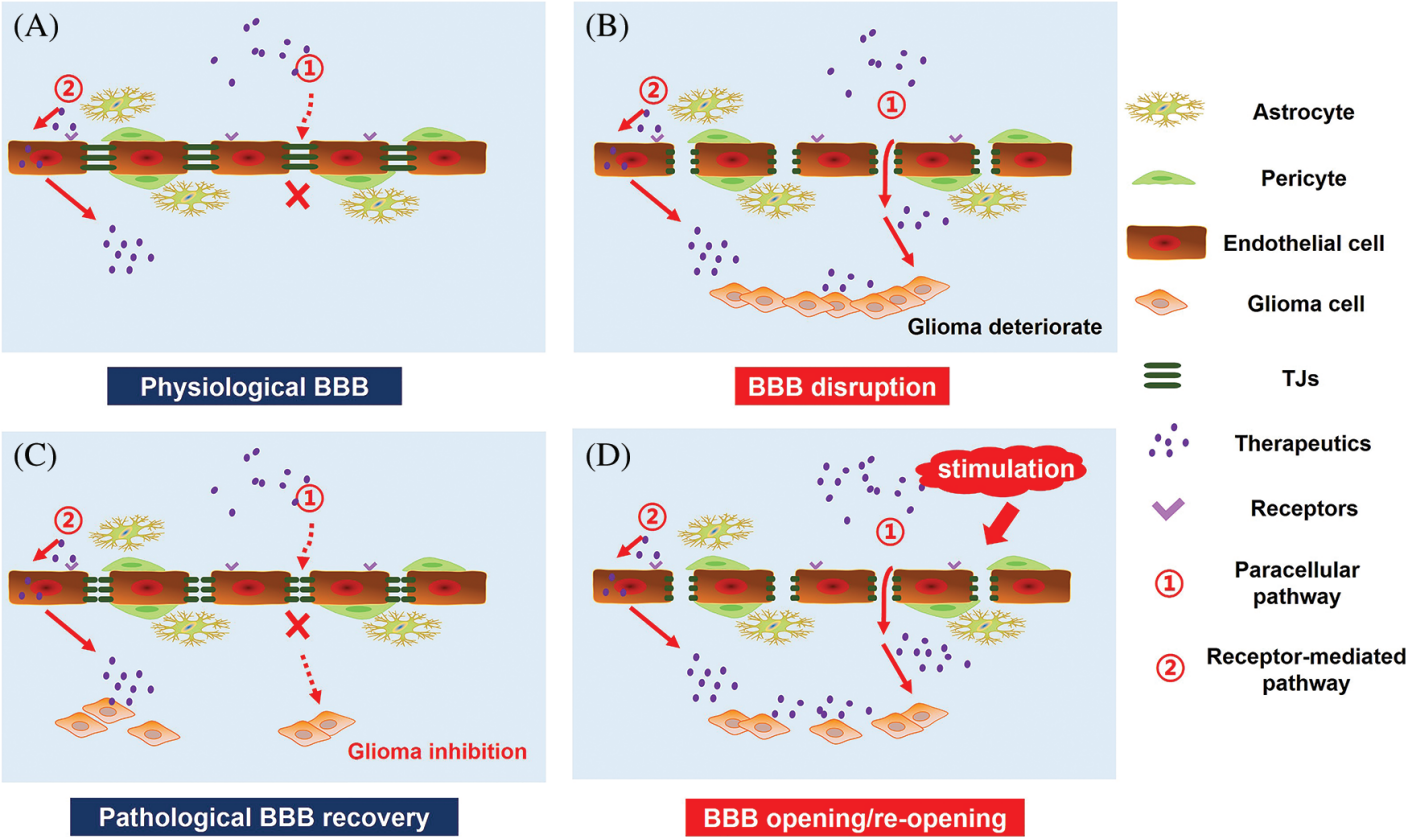
GBM therapy under the pathological state of the BBB. (A) Under physiological conditions, targeted therapeutics cross the BBB mainly by receptor-mediated pathway. (B) GBM growth causes BBB disruption and pathological fenestration, which in turn providing an extra paracellular way for therapeutics transporting into the brain. (C) Chemotherapy promotes pathological BBB functional recovery, which induces a significant decrease in targeted therapeutics transport across the BBB. (D) Opening of pathological BBB or re-opening of functional recovering pathological BBB via external stimulation, including biological stimuli, chemical stimuli, physical stimuli and signaling pathway regulators, could increase the intracranial delivery of targeted therapeutics for enhanced GBM therapy.

**Figure 3:**
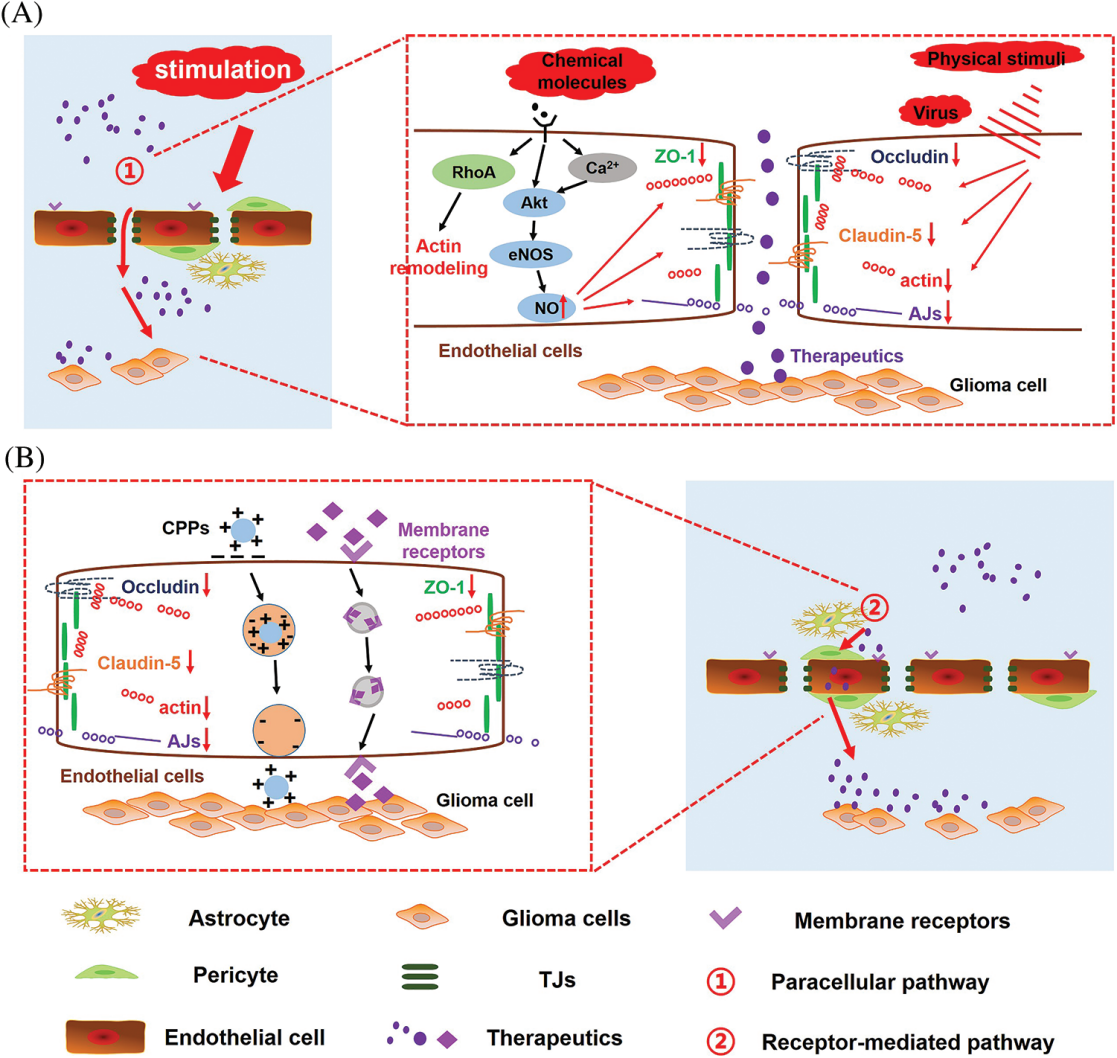
Mechanism of opening pathological BBB or re-opening functional recovering pathological BBB for enhanced drug delivery in the brain. Under biological stimuli, physical stimuli and chemical stimuli, (A) signaling pathways involved in the regulation of pathological BBB permeability and (B) transport routes of therapeutics across the BBB.

BBB permeability also exhibits high variability in different areas of the same tumor, as BBB integrity often exhibits high heterogeneity within tumor tissue. In the core of the cancer, the permeability of BBB is generally the strongest, while the barrier structure remains relatively intact in peripheral regions of the cancer. Moreover, the BBB of the peripheral GBM still exhibits highly functional. The intact BBB of tumor-infiltrating areas severely limits the effective delivery of chemotherapeutic drugs to GBM cells, and is a critical factor leading to the inefficiency of GBM treatment and high recurrence rate. Therefore, an essential strategy for the effective treatment of GBM is to increase the permeability of BBB and promote the intracerebral delivery of therapeutic drugs. Moreover, BBB pathological fenestration in GBM could provide an additional paracellular pathway for active targeting therapeutics transporting into the brain (Fig. 2B) [80–82]. At the same time, chemotherapy could promote the functional recovery of pathological BBB. Seriously, in the course of GBM multiple administration, the restoration of pathological BBB further limits the transport of intracerebral therapeutics, making anti-GBM therapy fail (Fig. 2C). Therefore, there has been much interest in seeking methods to further enhance endothelial permeability for biomedical and (nano-) pharmaceutics across the BBB in anti-GBM therapy (Fig. 2D). Here we reviewed three types of strategies opening pathological BBB for enhanced drug delivery in the brain (Fig. 3).

### Biological stimuli

The virus is a kind of biostimulant material that can open TJs via modulating chemokines as precursors for inflammatory cell infiltration in the CNS [83]. Verma et al. indicated that the West Nile virus exhibited the ability to cross the BBB via the “Trojan horse” mechanism by enhancing the expression of cell adhesion molecules, which allows macrophages to migrate through the BBB without disrupting the BBB [84]. Foust et al. also verified that adeno-associated virus (AAV9) exhibits practical ability to effectively target CNS cells, including neurons and adult astrocytes, without disrupting BBB integrity [85]. Rabies virus (RABV) is a highly neurotropic virus, the RABV glycoprotein expressed in RABV can specifically bind to nAchR on the surface of neuron cells to realize the intracerebral transport of the virus [70,86,87]. The unique properties of the virus crossing the BBB have led to a wide range of targeted therapies for brain diseases. Zhang et al. used recombinant adeno-associated virus rAAV as a carrier to introduce the single-chain antibody scFV targeting abnormal phosphorylated Tau protein (P-Tau) into the brain, successfully inducing the continuous expression of scFV *in vivo* for AD immunotherapy [88]. Qiao et al. used RABV as a biological carrier to wrap virus-like nanoparticles, which could effectively overcome BBB, finally realizing GBM targeting and safe treatment [89].

Although biological viruses can reach the brain in a reasonable and precise way, there are still some inherent security issues in the use of virus-mediated drug delivery systems. In addition, the existing virus-like biomimetic nanocarriers mostly focus on the simulation of specific viral proteins, while ignoring the physical and chemical properties of viruses, leading to low drug delivery efficiency and poor therapeutic efficacy. Therefore, the construction of biomimetic nanocarriers that are highly similar in structure, shape and function to the virus may be one of the most effective ways to target delivery therapeutics for brain diseases treatment in the future.

### Chemical stimuli

### Mannitol

Mannitol, an osmotic agent commonly explored, could shrink the endothelial cells line CNS capillaries with resultant separation of the endothelial TJs, which finally promotes passive diffusion of large molecules transport through the BBB. It has been used in combination with peptides, nanoparticles and gene delivery. Nevertheless, although mannitol therapy exhibits its beneficial effects on BBB opening, but there are still some risk factors, including brain damage, altered glucose uptake, the passage of plasma proteins and abnormal neuronal function [90,91]. Notably, mannitol produces non-selective disruption of the BBB, and only a 25% increase in the permeability of tumor microvasculature.

### Bradykinin

Bradykinin is a 9-peptide substance with a cardioprotective effect by reducing the infarct area of acute ischemia-reperfusion myocardium. Although the endogenous bradykinin may achieve some advances in regulating the permeability of BBB in animal studies, some drawbacks limit its application as a BBB permeability regulator, such as the short half-life (only several seconds), potent vasoactive metabolites [92], weak BBB opening effect. Therefore, the selective B2 bradykinin agonist Cereport (labradimil or RMP-7) has also been designed and investigated as potential inducer for BBB transient disruption. RMP-7 can cause intracellular Ca^2+^ influx by selectively binding to B2 receptors on the membrane of cerebral endothelial cells, which triggers a series of signal transmission reactions, including phosphatidylinositol, endothelial NOS and cGMP. The main mechanisms of RMP-7 in opening BBB is to increase the transport of endocytic vesicles or open the TJs of endothelial cells [93,94]. And it exhibits specific time-dependent and dose-dependent actions on human cerebral microvascular endothelial cells. Either intracarotid or i.v. administration with RMP-7 could produce an effect on BBB opening. Since RMP-7 cannot suppress the P-gp efflux pump, RMP-7 stimulation shows no effect on drugs that are P-gp substrates (such as paclitaxel) transported across the BBB.

### Alkylglycerols

Alkylglycerols can also be employed for BBB opening. The extent of disruption in the BBB mainly depends on the number and length of glycerols and alky groups presented in the chemical structure, respectively. Hulper et al. indicated that 1-O-pentylglycerol and 2-O-hexyldiglycerol exhibited the ability to reversibly increase the permeability of BBB, without any influence on the TJs strand complexity [95,96]. The phenomena could be due to protein rearrangement and alterations in the shape of the cells, and the cytoskeleton remodeling under the invention of alkylglycerols.

### ‘Orifice-opening’ agents

There are a series of traditional Chinese medicine (TCM) used explicitly for resuscitation purposes to restore consciousness in certain emergency conditions, such as coma, stroke, heart attack and brain-related traumatic brain injury etc. TCM resuscitation agents are mainly derived from aromatic minerals and animal materials, and are known as aromatic ‘orifice-opening’ agents. Borneol is a bicyclic monoterpene with fragrant odor, pungent and highly hydrophobic, which is a representative resuscitation agent in TCM that has been used in clinical practice for more than 1500 years. Increasing evidence prove that Borneol is an effective adjuvant that can modulate BBB permeability and promote drug delivery into the brain. The enhanced permeability induced by Borneol is closely associated with activating adenosine receptors, the down-regulation of TJs, the increase of void structures between the endothelial cells and the suppression of efflux protein function [97,98]. Moreover, systemic Borneol was found to modulate the vasodilatory neurotransmitters such as histamine and serotonin in the hypothalamus [99]. The activation of these neurotransmitters can induce cerebral vasodilation and enhance the permeability of BBB through nitric oxide and its receptors distributed on endothelial cells and astrocytes. However, current research is mainly limited to studies in cell lines or rodent-based models. There is still a lack of research data on permeation enhancing effect of Borneol in higher animal models such as pigs and primates. And the efficacy and safety of Borneol still need to be further studied.

### Adenosine

Adenosine, a purine nucleoside produced by many organs (heart, lung, gut, brain) and immune cells throughout the body, acts as a cellular metabolic distress signal. Its primary function is to reduce tissue damage and promote repair through diverse receptor-mediated mechanisms, including increasing oxygen supply ratio to demand, protecting against ischemic injury, anti-inflammatory effects and stimulating angiogenesis [100,101]. The combination of adenosine and A2A adenosine receptor (AR) expressed on the BBB could temporarily and transiently increase intercellular spaces between the brain endothelial cells, with an up-regulation of BBB permeability [102]. Kim et al. indicated that the activation of AR with Lexiscan increases the permeability of a primary human BBB with an increased transmembrane transport of chemotherapeutic drugs and T cells. And the phenomenon was mainly due to the morphological changes in actin-cytoskeletal reorganization induced by RhoA signaling and a potent down-regulated expression of VE-cadherin and Claudin-5 [103]. Gao et al. developed a series of nanoagonists (NAs) for AR activation, and NAs increased the permeability of the endothelial cell monolayer through TJs disruption. *In vivo* imaging studies further indicated that the intravenous injection of NAs could remarkably increase brain uptake of the model drug and the BBB opening time in a range of 0.5–2.0 h. And the temporary opening of the BBB induced by NAs shows the promise to reduce potential risks like overdosage and uncontrollable BBB leakage [104].

### 5-phosphodiesterase inhibitors

5-phosphodiesterase inhibitors are the selective inhibitors of type 5 PDE (PDE5), such as sildenafil, vardenafil, and tadalafil, which can prevent phosphodiesterases (PDE) from reducing intracellular cGMP levels down to 5′-GMP. cGMP is a crucial intracellular second messenger, which could activate cGMP-dependent protein kinase (PKG) and stimulate KCa channels to increase vesicle trafficking in brain tumor microvasculature [105,106]. Thus, as an oral treatment for erectile dysfunction in men approved by FDA, oral 5-phosphodiesterase inhibitors cannot regulate tight junction integrity in tumor capillary endothelium, but can increase transendothelial vesicular density to enhance the penetration of chemotherapy into the brain [107]. In addition, 5-phosphodiesterase inhibitors may reduce the chaperone glucose-regulated protein (GRP78) and P-glycoprotein involved in chemoresistance in glioblastoma mice and inhibit microglial activation induced by lipopolysaccharide (LPS), which indicated they were excellent drugs that can be combined with chemotherapy drugs to treat brain tumors [108,109].

### NOS-3 activators and NO donors

The nitric oxide synthase-3, (also called endothelial constitutive nitric oxide synthase, eNOS or ecNOS) was mainly expressed in endothelial cells and neurons. The principal physiological function of NOS-3 was to catalyze L-arginine to L-citrulline and produce NO with the participation of many cofactors, such as tetrahydrobiopterin (BH4) and calmodulin. Nitric oxide, as a biological messenger, was first identified as an endothelial cell relaxing factor and closely involved in mediating the regulation of the integrity of the BBB. On one hand, NO can activate soluble guanylate cyclase, resulting in increased intracellular cyclic GMP levels, further regulating the macromolecular transport into the brain microvessels [94]. For another, NO has an unpaired electron and readily reacts with H_2_O_2_ or proteins to form highly toxic peroxynitrite (ONOO^−^) and the S-nitrosylation proteins in some degenerative diseases or tumors [110]. Excessive ONOO^−^ and the S-nitrosylation of proteins severely damaged target cells and inactivated target enzymes, further enhancing the opening of the pathological blood-brain barrier [110,111].

### VEGF signaling pathway

VEGF-A is described as VPF (vascular permeability factor), a key regulator of normal and pathological blood vessel growth of the VEGF family, and a potent protein that rapidly and instantaneously enhances the vascular permeability of an intact BBB [112]. It is widely acknowledged that VEGF-A possesses two major biological activities, one is to stimulate the proliferation of vascular endothelial cells, the other is to increase vascular permeability [113]. The increase in vascular permeability induced by VEGF-A is common in pathological angiogenesis areas, such as solid tumors, wounds, and chronic inflammation. Notably, although some inflammatory cytokines, such as histamine and bradykinin, can also induce increased vascular permeability, experimental evidence showed that vascular leakage caused by VEGF and inflammatory mediators occurs through different molecular processes [114]. Microvascular wall exposure to VEGF induces permeability with abnormally rapid kinetics and 50,000 times higher than histamine [115].

The mechanism of VEGF-A in the regulation of vascular permeability is mainly involved with several complex transmembrane transport processes [115]. VEGF-A could induce fenestrations for small solute leakage, and also cause the formation of caveolae, which assembled into vesiculovacuolar organelles (VVOs) for protein transmembrane transport. Leakage of larger proteins and extravasation of erythrocytes depends on the release of vascular endothelial (VE), which mediates adhesions by cadherin. Furthermore, VEGF-A phosphorylates VEGFR2 and activates downstream signaling pathways involved in permeability regulation process. VEGF regulates the production of endothelial nitric oxide synthetase (eNOS) by PI3K/AKT. Furthermore, the activation of VEGFR2 stimulated phospholipase C-γ(PLC), produced diacylglycerol (DAG) and PKC, which possibly activated the nonselective cation channels (e.g., TRPC6 or TRPC3) and induced calcium influx [116].

### Physical stimuli

### Ultrasound-facilitated opening

Focused ultrasound (FU) is emerging as a new strategy for localized, reversible, and safe BBB disruption to enhance therapeutics delivery to the brain [117]. However, due to the complex structure of the brain, FU is easily reflected by the skull, which significantly attenuates the ultrasonic energy, and brings new challenges to the use of ultrasound to open the BBB for the treatment of brain diseases. Therefore, FU is typically used with prefabricated microbubbles (FU-MBs) consisting of albumin or lipid core, which encapsulate a semi-solid gas (perfluorocarbon) and are confined in the vasculature. FU-MBs can realize the non-invasive, targeted and reversible opening BBB at a low sound pressure, with good repeatability and no permanent damage to brain tissue. At present, FU-MBs has been widely used in delivering nano drug delivery system, gene drugs and other small molecule drugs across the BBB [59,118,119]. The potential mechanisms for intracerebral transport induced by FU-MBs include transcytosis, partial opening of interendothelial clefts and TJs, transendothelial channel formation and fenestration, and free passage through the damaged endothelium [120,121]. However, the use of FU-MBs may be accompanied by the temperature increase of local target tissues, resulting in microvascular rupture and cerebral edema. In addition, the type and concentration of MBs would seriously affect the degree and duration of ultrasonic opening of the BBB. Therefore, optimizing ultrasound equipment, controlling ultrasound parameters (sound pressure, frequency, irradiation mode, etc.) and screening appropriate MBs doses have important guiding significance for FU-MBs to mediate BBB opening in the treatment of brain diseases and clinical translation.

### Microwave-facilitated opening

Microwave is another physical stimulus to open BBB for enhancing drug delivery into the brain. Wang et al. found that microwave exposure could cause VEGF/FlK-1-ERK pathway activation and Occludin phosphorylation, accompanied by Occludin down-regulation and its interaction with ZO-1 [122]. In addition, the resultant brain temperature increase induced by sufficiently strong microwave energy may also produce increased BBB permeability. Indeed, previous study has reported that the rate head exposed to microwave frequencies (2.5–3.2 GHz) could make brain temperature rise with an enhanced permeability of HRP, Evans blue and sodium fluorescein to BBB, and the temperature measured above 40°C. When brain temperature cooled below 40°C, the BBB opening effect is ignorable, suggesting that the mechanism of BBB opening is also related to the thermal effect of microwaves [123,124]. Notably, the thermal effect induced by microwaves may also increase brain infections. Furthermore, long-term exposure to microwave radiation can easily cause damage to brain tissues, resulting in symptoms such as memory loss, insomnia, headaches and dreams. The related mechanisms remain unclear, possibly including neuronal degeneration, apoptosis and necrosis, mitochondria swelling and cavitation, reduction of Nissl bodies, BBB damage, synaptic structure and function of plastic damage and calcium overload [125]. For safety concerns, there are no relevant reports on the application of microwave-opening BBB in treating brain diseases.

### Electromagnetic field

The use of electromagnetic field (EMF) pulses can also increase BBB permeability. Qiu et al. reported that EMF pulses could regulate protein kinase C signaling and ZO-1 translocation, inducing BBB permeability to increase [18]. Experimental data *in vitro* further showed enhanced transportation of antiviral drugs across BBB with EMF stimuli, and the wave shape, frequency and amplitude of EMF directly affect the intracerebral delivery efficiency of drugs [126]. Do et al. also confirmed that applying an external EMF could significantly enhance the rate of nanoparticles across the BBB [127]. Nano-carrier combined with EMF enormously facilitates intracerebral delivery of therapeutic drugs and could have potential clinical application in treating brain tumors and other brain-related diseases.

## Conclusions and Prospects

So far, no effective treatments for brain diseases (AD, PD, stroke, etc.), including GBM. In addition to the complexity of these diseases, the main reason is the presence of the BBB. For decades, the delivery of drugs to the brain and spinal cord has been an exciting but exhausting research area due to the highly complex structure of the BBB. With the development of pharmaceutical technology, therapeutic pharmaceutics and methods aiming at the BBB are constantly being discovered. Controllably regulating BBB opening provides the possibility for the transport of medicinal drugs into the brain. Various drug transport systems (AMT, CMT, RMT) also show great potential in the transportation of biological macromolecules and small molecules across the BBB. The emergence of biomimetic nanotechnology makes the treatment of brain diseases safer and more efficient.

BBB dysfunction, often referred to as “BBB opening,” is likely to be the common feature of the progression of brain diseases, including GBM. One practical and rational way to design a novel drug delivery system for intracerebral therapeutics delivery is to study the molecular mechanism regulating BBB permeability, and further utilize the differences between physiological conditions and pathological conditions. Notably, the brain is a vital organ that governs all the life activities of the human body. It is also necessary to evaluate the safety, risk and benefit for patients when the new technologies or brain-targeted systems are used to increase BBB permeability, especially for some brain diseases requiring long-term drug treatment. Overall, in the design of an intracerebral drug delivery system, significantly to increase the penetration of drugs in the brain, it is necessary to consider the selectivity of drugs for the lesion site, minimizing the distribution in non-target tissues.
